# Effect of death education interventions on death attitudes in medical students: systematic review and meta-analysis

**DOI:** 10.3389/fpubh.2025.1754182

**Published:** 2026-02-04

**Authors:** Nan Wang, Zhizhong Wang

**Affiliations:** 1 School of Humanities and Social Sciences, Shanxi Medical University, Taiyuan, Shanxi, China; 2 First Hospital of Shanxi Medical University, Taiyuan, Shanxi, China

**Keywords:** death attitude, death education, medical students, meta-analysis, PRISMA, Stata

## Abstract

**Background:**

The quality of palliative care depends on medical practitioners’ death cognition and care ability, however, medical students worldwide generally face problems such as death anxiety and insufficient palliative communication skills. Existing studies have not yet formed systematic conclusions on the consistency of the effects of death education interventions and the optimal intervention model.

**Methods:**

Following PRISMA, 5 databases (PubMed, Web of Science, Cochrane Library, CNKI, Wanfang Data) were searched (2010.1–2025.10), including 12 non-randomized intervention studies (1,111 students). Quality was assessed using the Methodological Index for Non-Randomized Studies Scale (MINORS). Stata 18.0 conducted meta-analysis, with subgroup (intervention method, duration, culture), sensitivity, and publication bias (funnel plot, Egger’s test) analyses.

**Results:**

Meta-analysis showed that death education interventions (based on non-randomized intervention studies evidence) had potential positive effects: it reduced fear of death (SMD = −0.38, 95%CI [−0.59, −0.17]), death avoidance (SMD = −0.40, 95%CI [−0.64, −0.17]), and escape acceptance (SMD = −0.32, 95%CI [−0.58, −0.06]); meanwhile, it enhanced neutral acceptance (SMD = 0.43, 95%CI [0.15, 0.72]) and approach acceptance (SMD = 0.29, 95%CI [0.01, 0.58]). Additionally, preliminary results showed that death education interventions improved attitudes toward palliative care. Exploratory subgroup analysis revealed that: blended learning had the promising effect; interventions with a duration of ≥16 class hours showed better improvement; Chinese students exhibited significant improvement in neutral acceptance, while others performed better in approach acceptance. It should be noted that several subgroup analyses include small numbers of studies and are explicitly labeled as exploratory. Sensitivity analysis indicated that the pooled effect size had a small fluctuation range. The funnel plot and Egger’s test suggested no significant publication bias. Pooled analyses of all DAP-R dimensions show very high heterogeneity (I^2^ > 80%), which may affect the reliability and generalizability of the pooled effect sizes.

**Conclusion:**

Caution is warranted in causal interpretation given the non-randomized nature of included studies. Death education interventions shows potential improving medical students’ death attitudes and enhancing their palliative care literacy. Blended learning and ≥16 class hours might be more effective. It is suggested that medical colleges integrate it into core curricula, optimize design per culture, and build long-term follow-up systems to achieve “humanity-technology integration.”

## 1 Introduction

As an inevitable end of human life, death remains a sensitive topic in many cultures. Particularly in China, the Confucian traditional values of “valuing life and avoiding death” have formed a deeply rooted death taboo (1). This cultural context, combined with the lack of death education in medical and nursing education systems, results in insufficient preparedness among many medical students when facing death in clinical practice. An integrated analysis of 9,749 nursing students from 13 countries found that although students generally held positive attitudes toward palliative care, their knowledge level was significantly insufficient (2). Among Saudi Arabian nursing students, 86.6% had no experience of systematic death education (3); in China, 75.55% of medical students reported being “very unfamiliar” or “unfamiliar” with death-related knowledge (4). Similarly, a survey of 907 undergraduate nursing students in central and western China showed that 76.9% of the students had a clear demand for death education, but only 15% of medical colleges and universities included palliative care in their compulsory courses, and most nursing majors allocated less than 8 h to palliative care content (5).

Currently, there is a widespread lack of a standardized framework for palliative care competency development in nursing education in the Asia-Pacific region (6). In terms of intervention models, constructivist courses featuring “group discussions + role-playing + immersive experiences” have improved students’ mastery of palliative care knowledge and their willingness to apply it (7). Simulated death experience is an emerging intervention method in recent years (8); in addition, “simulated palliative communication” training for medical students has confirmed that using standardized patients to simulate real death scenarios can significantly enhance students’ communication skills and emergency response capabilities (9).

Although existing studies have confirmed that death education can improve medical students’ death attitudes (10), and nurses who have received systematic death education demonstrate significantly better abilities in pain management, patient communication, and family comfort compared to those who have not (11), medical students without death education are more likely to experience negative emotions such as helplessness and anxiety when facing patient death, and may even develop occupational burnout and turnover intention (12). However, the differences in effects among different intervention models (e.g., theoretical teaching, practical experience, blended learning) remain unclear, and there is a lack of systematic evaluation conclusions on whether there is heterogeneity in the intervention effects across various dimensions of the Death Attitude Profile-Revised (DAP-R) (13), such as fear of death and neutral acceptance. Previous studies have mostly focused on a single major (e.g., nursing) or a single region, resulting in limited sample representativeness, and have not deeply explored the moderating effects of factors such as intervention duration and cultural background on the outcomes (14, 15).

Based on this, this study systematically searched Chinese and English literatures and used meta-analysis to integrate existing evidence, aiming to clarify the overall effect of death education on medical students’ death attitudes and key influencing factors, as well as the optimal intervention program. This study provides a scientific basis for the reform of medical education curricula.

## 2 Methods

### 2.1 Study design

This meta-analysis strictly adheres to the PRISMA statement (16), and the original data can be obtained by contacting the corresponding author. All included studies are before-and-after controlled or quasi-experimental designs, with no randomized controlled trials, and the evidence base of this study is derived from non-randomized intervention studies.

### 2.2 Literature search strategy

The searched databases included PubMed, Web of Science, Cochrane Library, CNKI, and Wanfang Data, with a retrieval period from January 1, 2010, to October 31, 2025 (since death education-related studies after 2010 have more standardized designs and are more in line with current medical education needs). For online-ahead-of-print articles within this period, only those that had undergone peer review were included (unreviewed preprints were excluded), ensuring the timeliness, comprehensiveness, and academic rigor of the included evidence. Chinese search terms: “medical students,” “death education,” “hospice care,” “palliative care”; English search terms: “medical students,” “death education,” “palliative care education,” “end-of-life care training.” A combination of “subject terms + free words” was used for retrieval. Meanwhile, the reference lists of included literatures were manually searched to supplement relevant studies that were not detected, avoiding omissions.

### 2.3 Inclusion and exclusion criteria

#### 2.3.1 Study type

Published before-and-after controlled trial studies on death education in Chinese or English; reviews, case reports, and studies with only qualitative results were excluded.

#### 2.3.2 Study participants

Full-time medical students (including nursing, clinical medicine, pharmacy, rehabilitation, etc.); participants with mental illness or major bereavement experience in the past 6 months were excluded.

#### 2.3.3 Intervention measures

Participants received explicit death education interventions, including but not limited to: palliative care courses, death experience activities (e.g., simulated death, funeral home visits), and online death education courses.

#### 2.3.4 Outcome measures

##### 2.3.4.1 Primary outcome measure

Medical students’ death attitudes, assessed using the Death Attitude Profile-Revised (DAP-R) scale (13). This scale consists of 32 items, divided into 5 dimensions: Fear of Death (7 items), Death Avoidance (5 items), Neutral Acceptance (5 items), Escape Acceptance (10 items), and Approach Acceptance (5 items). The interpretation of each dimension is as follows: Fear of Death (higher score indicates stronger fear of death), Death Avoidance (higher score indicates stronger tendency to avoid death), Neutral Acceptance (higher score indicates a stronger tendency to view death as a natural physiological process), Approach Acceptance (higher score indicates a stronger belief in exploring the meaning of life through death), and Escape Acceptance (higher score indicates a stronger tendency to view death as a means to escape pain).

##### 2.3.4.2 Secondary outcome measure

Attitudes toward palliative care, assessed using the Frommelt Attitude Toward Care of the Dying (FATCOD) scale (17). The FATCOD is a 30-item questionnaire (15 positive and 15 negative statements) used to evaluate attitudes toward caring for terminally ill patients and their families. Before analysis, negative items were reverse-scored. A higher score indicates a more positive attitude.

#### 2.3.5 Exclusion criteria

(a) Studies involving non-medical students (e.g., psychology, sociology majors); (b) Duplicate publications, literatures with incomplete data or inability to extract effect sizes (e.g., only *p*-values reported without means and standard deviations); (c) Studies that include death education in interventions but cannot separately analyze its effect (e.g., mixed multiple interventions with inability to separate the effect of death education).

### 2.4 Data extraction and quality assessment

#### 2.4.1 Data extraction

Two researchers independently conducted literature screening and data extraction, with discrepancies resolved through consultation with a third researcher. The extracted content included: author, year, country/region, study type, sample size, intervention protocol, course duration, outcome indicators (means and standard deviations of each DAP-R dimension and secondary outcomes), and quality-related information (random sequence generation method, allocation concealment measures, blinding implementation).

#### 2.4.2 Quality assessment

The Methodological Index for Non-Randomized Studies (MINORS) scale was used for quality assessment (18). The scale includes 12 items, scored as 0 points (not reported), 1 point (reported but insufficient), or 2 points (reported and sufficient), with a total score of 24. Studies with a score ≥16 were considered high-quality. It must be emphasized that high MINORS scores do not equate to high-level evidence comparable to randomized trials, as the inherent limitations of non-randomized studies (such as potential selection bias) cannot be completely eliminated by this scale.

### 2.5 Statistical analysis

Stata 18.0 software was used for statistical analysis, following these steps:

- Effect size calculation: the standardized mean difference (SMD) was used to pool effect sizes (19). According to Cohen’s criteria: |SMD| 0.2–0.5 = “small effect,” 0.5–0.8 = “medium effect,” >0.8 = “large effect” (20).
- Heterogeneity test: the I^2^ statistic and Q-test were used to assess heterogeneity. I^2^ < 25% indicates low heterogeneity, 25% ~ 50% indicates moderate heterogeneity, and >50% indicates high heterogeneity. A fixed-effects model was used for pooling when there was low/moderate heterogeneity, and a random-effects model was used when there was high heterogeneity (21). Subgroup analyses (based on intervention methods, course duration, and cultural background) were conducted to explore the sources of heterogeneity.
- Publication bias assessment: a funnel plot was used for visual judgment of publication bias. If the funnel plot was asymmetric, Egger’s test was further used for quantification (*p* < 0.05 indicates significant publication bias) (22).
- Sensitivity analysis: the “one-study-at-a-time exclusion” method was used (23). The pooled effect size was recalculated after excluding each study one by one, and the fluctuation range was observed to verify the stability of the results.

## 3 Results

### 3.1 Literature selection process

**Figure 1:**
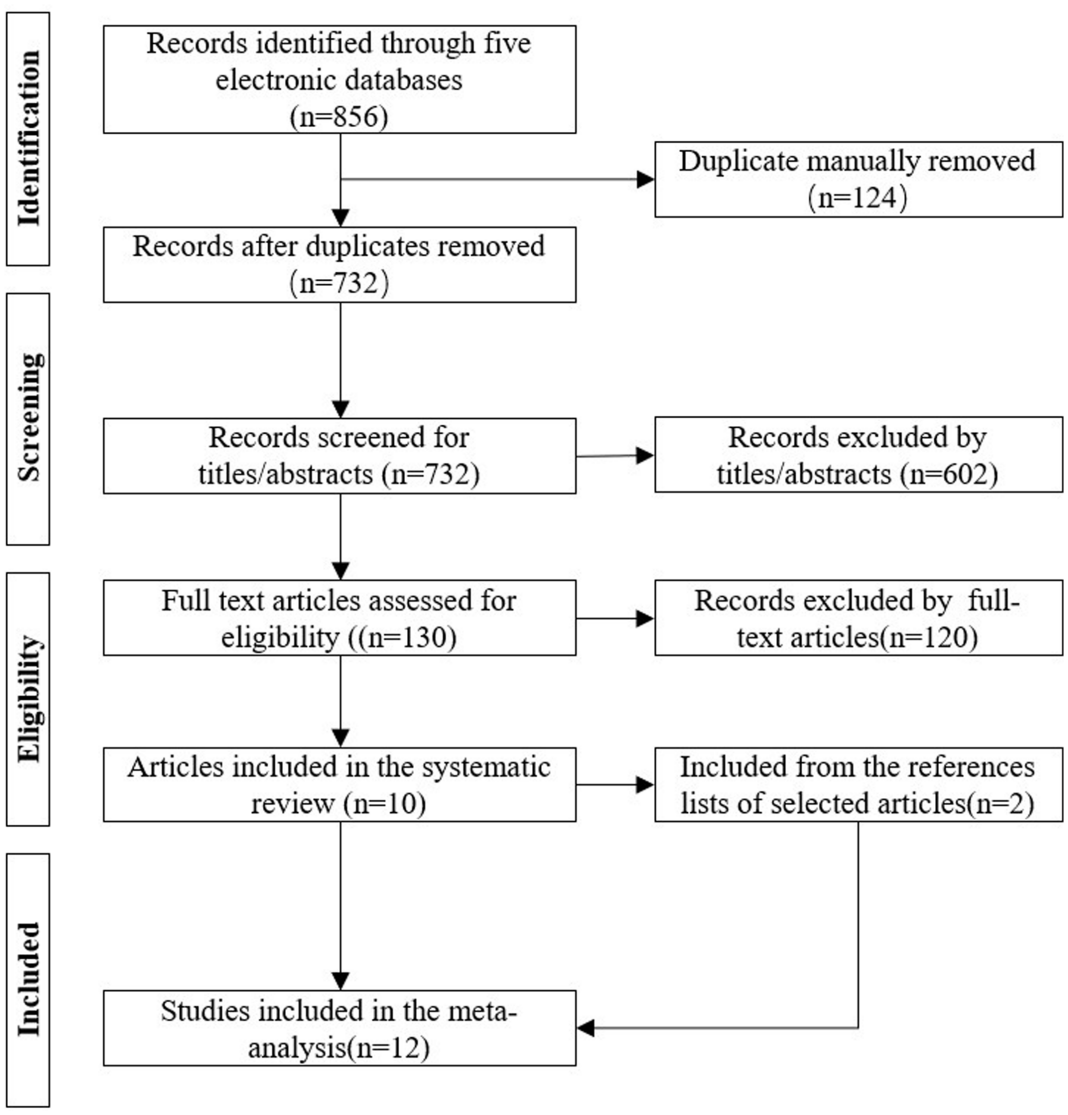
Literature selection flowchart (drawn in accordance with the PRISMA statement).

Initially, 856 literatures were retrieved. After removing 124 duplicate literatures using EndNote 2025, 602 irrelevant literatures were excluded by reading titles and abstracts. A total of 130 literatures were further assessed for eligibility by full-text reading, among which 120 were excluded for not meeting the inclusion criteria. Two additional studies were included from the reference lists of selected articles. Finally, 12 studies were included in the systematic review, including 6 English (3, 14, 15, 24–26) and 6 Chinese (4, 27–31) literatures, involving 1,111 medical students. The PRISMA flow diagram is shown in Figure 1.

### 3.2 Characteristics of included studies

The 12 included studies involved 1,111 medical students, covering majors such as nursing, clinical medicine, pharmacy, and rehabilitation. The study regions included China (8 studies), Turkey (2 studies), the United States (1 study), and Saudi Arabia (1 study). The intervention methods were categorized into 3 types: offline (8 studies), online (1 study), and blended learning (3 studies, e.g., online MOOCs + offline simulation). The intervention duration ranged from 2 to 32 class hours. The outcome indicators mainly included the DAP-R scale (12 studies) and the FATCOD scale (3 studies). All 12 studies had a MINORS score ≥16 (high quality). Detailed characteristics are presented in Table 1.

**Table 1:** Basic characteristics of included studies.

Included study	Country/region	Sample size	Intervention protocol	Course duration	Outcome indicators	Quality score (MINORS)
Chen et al. (27)	China	105	Blended learning (online videos + offline simulation)	32 class hours	DAP-R, Course Satisfaction	19
Conner et al. (14)	USA	58	Online death education (videos + forums + reflection)	16 weeks	DAP-R, FATCOD	20
Huang et al. (28)	China	205	Blended death education (online theory + offline discussion)	32 class hours	DAP-R	18
Huang et al. (29)	China	64	Balint Group (end-of-life case sharing + group support)	2 class hours	DAP-R, JSE	18
Ibrahim et al. (3)	Saudi Arabia	216	Video courses + virtual cases + offline discussion	12 weeks	DAP-R, FATCOD, PCQN	18
Karaca and Ercan Sahin (15)	Turkey	53	Spiritual care education + end-of-life case analysis	28 class hours	DAP-R, SSCRS	16
Özveren et al. (24)	Turkey	94	Storytelling + case discussion + reflection diary	14 weeks (28 class hours)	DAP-R	16
Sun et al. (4)	China	45	Student-centered death education (thematic debate)	13 weeks (29 class hours)	DAP-R	16
Wang et al. (30)	China	80	Palliative care elective course (symptom management + grief counseling)	18 class hours	DAP-R, C-MLQ	16
Xu et al. (36)	China	65	Life care social practice (hospice visits)	32 class hours	DAP-R, CAI	20
Yang et al. (25)	China	66	Four-stage constructivist teaching (listening-seeing-touching-transcending)	28 class hours	DAP-R, Course Satisfaction	17
Zhu et al. (26)	China	60	Narrative teaching (movies + literature + role-playing)	6 class hours	DAP-R, CDS, FATCOD	18

DAP-R = Death Attitude Profile-Revised; PCQN = Palliative Care Quiz for Nurses; JSE = Jefferson Scale of Empathy; FATCOD = Frommelt Attitude Toward Care of the Dying Scale; C-MLQ = Chinese Version of Meaning in Life Questionnaire; SSCRS = Spiritual Care Competence Rating Scale; CAI = Care Ability Inventory.

### 3.3 Meta-analysis results

#### 3.3.1 Death attitudes (DAP-R scale dimensions)

**Figure 2:**
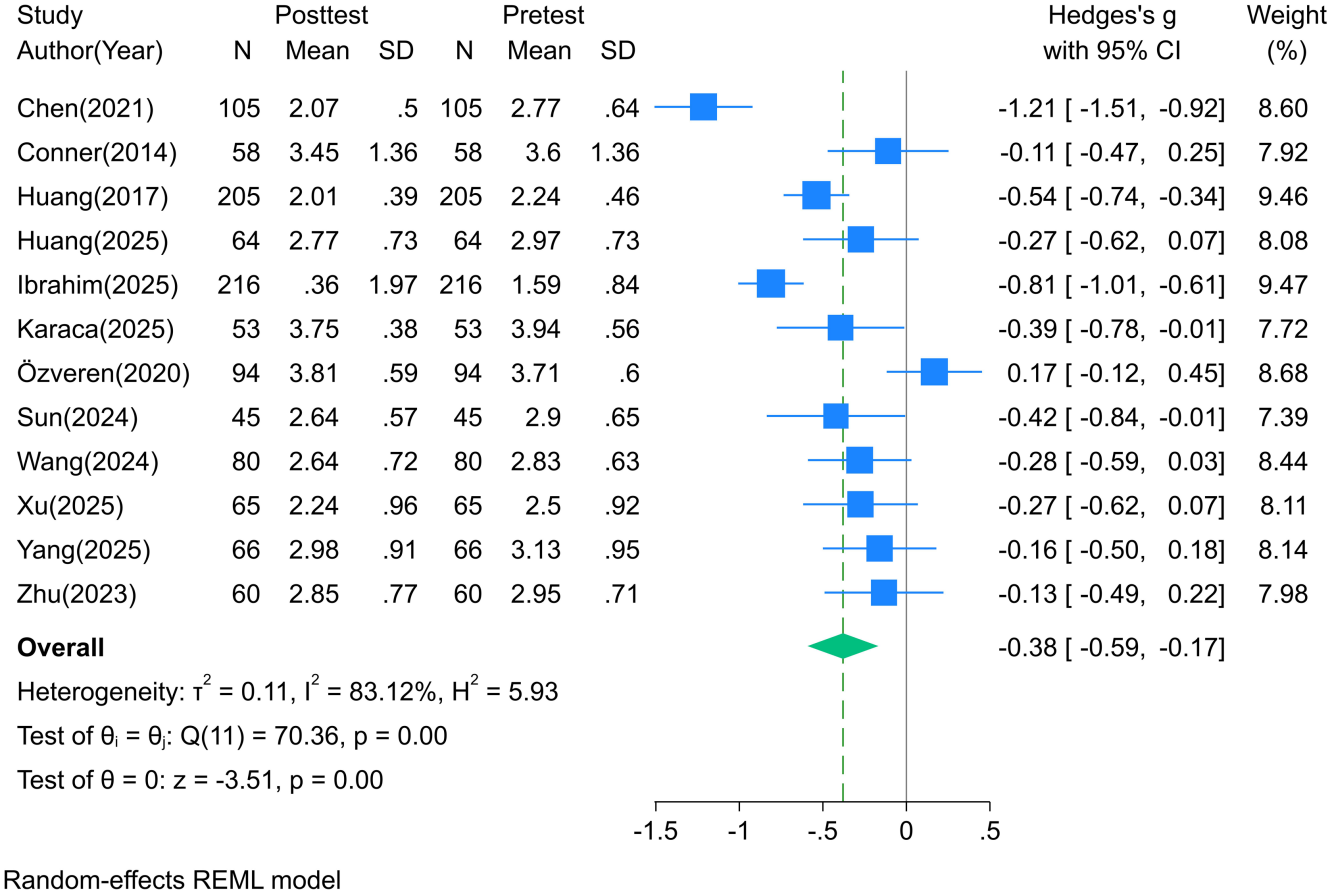
Forest plot of the effect of death education on the fear of death dimension.

All 12 studies reported DAP-R scale data. The heterogeneity test showed I^2^ > 50% (83.12% ~ 90.79%), so a random-effects model was used for analysis. A forest plot for the fear of death dimension is shown in Figure 2, and the pooled effect sizes for each dimension are presented in Table 2.

- Fear of death: the educational intervention significantly reduced individuals’ fear of death, with a pooled SMD = −0.38 (95%CI [−0.59, −0.17], *p* < 0.001) (medium effect), indicating that education can effectively alleviate anxiety and fear toward death.
- Death avoidance: the intervention also significantly improved death avoidance, with a pooled SMD = −0.40 (95%CI [−0.64, −0.17], p < 0.001) (medium effect), suggesting that education helps reduce the tendency to cope with death through avoidance or denial, and promotes rational confrontation with death-related topics.
- Neutral acceptance: the intervention significantly enhanced individuals’ neutral acceptance of death, with a pooled SMD = 0.43 (95%CI [0.15, 0.72], *p* < 0.001) (medium effect), indicating that death education interventions can help individuals establish a neutral and objective attitude toward death.
- Approach acceptance: the intervention had a significant positive effect on approach acceptance, with a pooled SMD = 0.29 (95%CI [0.01, 0.58], *p* = 0.04) (small to medium effect), demonstrating that education can to some extent enhance individuals’ positive tendency to explore the meaning of life through death and strengthen the sense of existential meaning.
- Escape acceptance: the intervention significantly reduced escape acceptance, with a pooled SMD = −0.32 (95%CI [−0.58, −0.06], *p* = 0.02) (medium effect), indicating that education can effectively reduce the negative cognition of viewing death as an escape method and guide individuals to view the relationship between death and life in a healthier way.

**Table 2:** Basic characteristics of pooled effect sizes for each DAP-R dimension.

Study	Fear of death	Death avoidance	Neutral acceptance	Approach acceptance	Escape acceptance
Chen et al. (27)	−1.21 [−1.51, −0.92]	−1.08 [−1.37, −0.79]	0.26 [−0.01, 0.53]	0.03 [−0.24, 0.3]	−0.92 [−1.21, −0.64]
Conner et al. (14)	−0.11 [−0.47, 0.25]	−0.25 [−0.62, 0.11]	0.15 [−0.21, 0.51]	0.06 [−0.3, 0.42]	0.2 [−0.16, 0.56]
Huang et al. (28)	−0.54 [−0.74, −0.34]	−0.31 [−0.51, −0.12]	0.41 [0.22, 0.61]	0.25 [0.05, 0.44]	−0.1 [−0.29, 0.09]
Huang et al. (29)	−0.27 [−0.62, 0.07]	−0.16 [−0.5, 0.19]	0.53 [0.18, 0.88]	−0.03 [−0.37, 0.32]	−0.21 [−0.56, 0.13]
Ibrahim (3)	−0.81 [−1.01, −0.61]	−0.95 [−1.15, −0.75]	1.56 [1.34, 1.77]	1.71 [1.49, 1.93]	−0.87 [−1.06, −0.67]
Karaca and Ercan Sahin (15)	−0.39 [−0.78, −0.01]	−1.05 [−1.45, −0.64]	1.33 [0.91, 1.75]	0.55 [0.17, 0.94]	−1.36 [−1.78, −0.94]
Özveren et al. (24)	0.17 [−0.12, 0.45]	0.2 [−0.09, 0.48]	−0.05 [−0.33, 0.24]	0.34 [0.06, 0.63]	−0.02 [−0.3, 0.27]
Sun et al. (4)	−0.42 [−0.84, −0.01]	−0.42 [−0.83, 0]	0.37 [−0.04, 0.79]	−0.04 [−0.45, 0.37]	−0.09 [−0.5, 0.32]
Wang et al. (30)	−0.28 [−0.59, 0.03]	−0.14 [−0.45, 0.17]	0.01 [−0.3, 0.32]	0.09 [−0.22, 0.4]	−0.09 [−0.4, 0.22]
Xu et al. (36)	−0.27 [−0.62, 0.07]	−0.25 [−0.59, 0.09]	0.13 [−0.21, 0.47]	0.17 [−0.18, 0.51]	−0.23 [−0.57, 0.11]
Yang et al. (25)	−0.16 [−0.5, 0.18]	−0.28 [−0.62, 0.06]	0.21 [−0.13, 0.55]	0.05 [−0.29, 0.39]	−0.14 [−0.48, 0.2]
Zhu et al. (26)	−0.13 [−0.49, 0.22]	−0.15 [−0.51, 0.2]	0.27 [−0.09, 0.63]	0.24 [−0.12, 0.59]	0.01 [−0.34, 0.37]
Pooled Effect Size	−0.38 [−0.59, −0.17]	−0.40 [−0.64, −0.17]	0.43 [0.15, 0.72]	0.29 [0.01, 0.58]	−0.32 [−0.58, −0.06]

#### 3.3.2 Attitudes toward palliative care (FATCOD scale)

**Figure 3:**
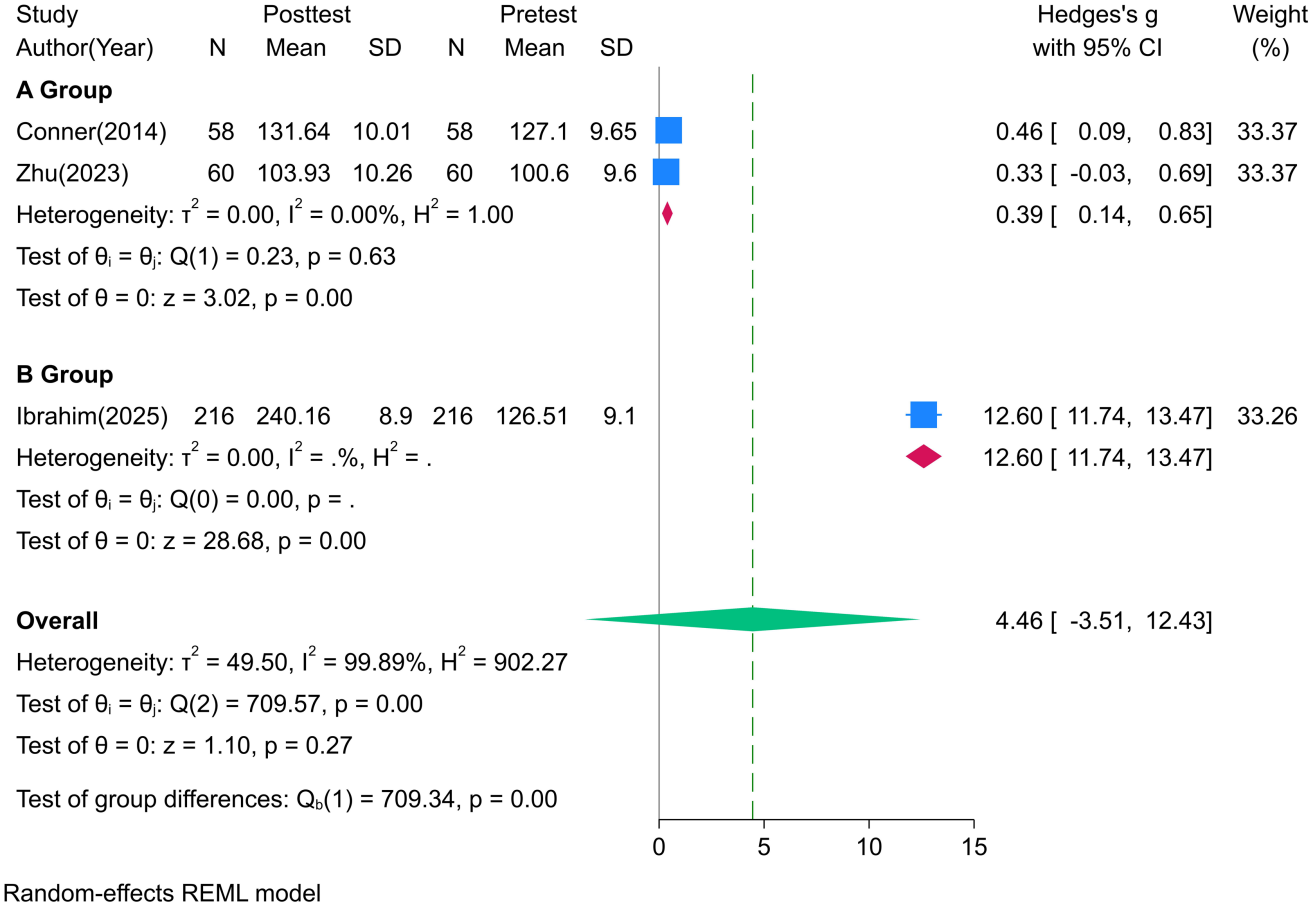
Forest plot of the effect of death education on attitudes toward End-of-Life care.

Three studies reported data on attitudes toward palliative care. However, the scoring method of (3) was consistent with the standard FATCOD scale, but the final score exceeded the theoretical maximum score of 150, which may be due to data entry errors or other measurement biases. To maintain consistency in data synthesis, only the pooled effect size of Subgroup A was analyzed (0.39 [0.14, 0.65]). As shown in Subgroup A of Figure 3, death education interventions significantly improved students’ positive attitudes toward palliative care (strong effect), such as proactive participation in communicating with terminally ill patients and emphasis on grief support for family members. Given the very small number of studies contributing to this analysis (*n* = 2), this finding must be regarded as preliminary and hypothesis-generating.

### 3.4 Subgroup analysis

To explore the sources of heterogeneity, subgroup analyses were conducted based on “intervention methods,” “course duration,” and “cultural background.”

#### 3.4.1 Subgroup by intervention methods

**Figure 4:**
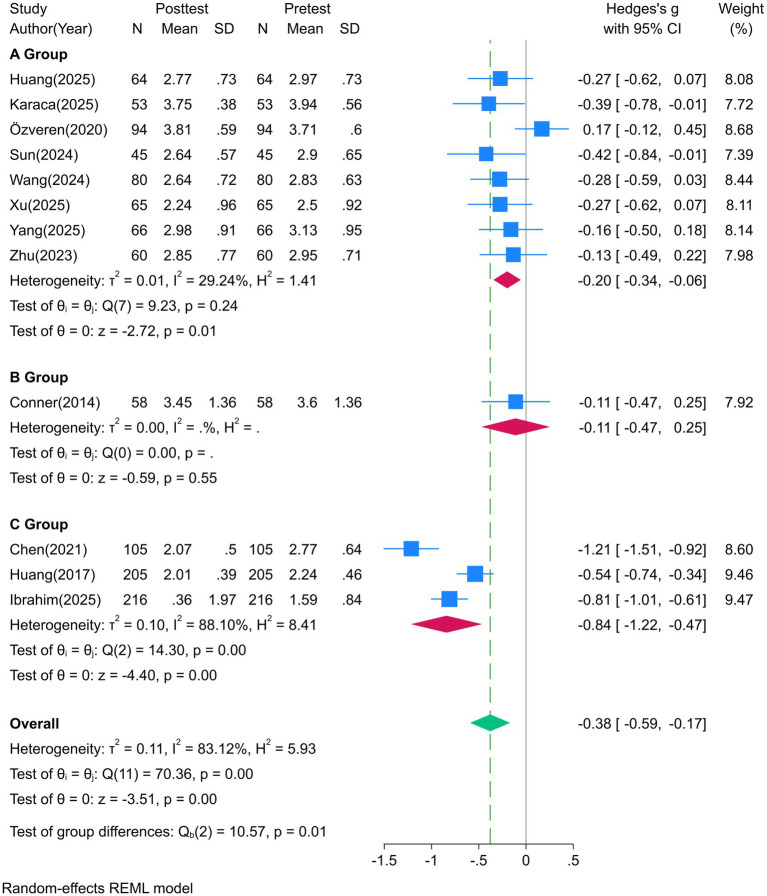
Forest plot of the effect of death education on the fear of death dimension by intervention Method (Group A: Offline; Group B: Online; Group C: Blended).

Intervention methods were categorized into 3 types (offline, online, blended). Table 3 presents the corresponding effect sizes from subgroup analyses grouped by intervention type, and Figure 4 shows the forest plot for the effect of death education interventions on the fear of death dimension. The results (Table 3) showed that blended learning had the optimal effect in improving death attitudes (fear of death: SMD = −0.84, 95%CI [−1.22, −0.47], *p* < 0.001, medium-large effect; Figure 4), followed by offline learning (fear of death: SMD = −0.20, 95%CI [−0.34, −0.06], *p* = 0.01, small effect), and online learning had the weakest effect (fear of death: SMD = −0.11, 95%CI [−0.47, 0.25], *p* = 0.55, no effect). This suggests that courses should integrate online and offline blended learning. These subgroup findings are exploratory due to the small number of studies in some categories (e.g., only 1 online learning study) and should be interpreted as hypothesis-generating rather than definitive evidence.

**Table 3:** Effect sizes of subgroup analysis by intervention methods.

Subgroup (intervention method)	Offline	Online	Blended
Number of studies	8	1	3
Sample size	527	58	526
Fear of death	−0.20 [−0.34, −0.06]	−0.11 [−0.47, 0.25]	−0.84 [−1.22, −0.47]
*p*-value	0.01	0.55	<0.001
Death avoidance	−0.27 [−0.50, −0.03]	−0.25 [−0.62, 0.11]	−0.78 [−1.24, −0.31]
*p*-value	0.03	0.17	<0.001
Neutral acceptance	0.02 [−0.36, 0.41]	0.15 [−0.21, 0.51]	0.74 [−0.06, 1.55]
*p*-value	0.90	0.42	0.07
Approach acceptance	0.18 [0.05, 0.30]	0.06 [−0.30, 0.42]	0.66 [−0.37, 1.70]
*p*-value	0.01	0.74	0.21
Escape acceptance	−0.25 [−0.55, 0.04]	0.20 [−0.16, 0.56]	−0.62 [−1.15, −0.10]
*p*-value	0.09	0.28	0.02

#### 3.4.2 Subgroup by course duration

Courses were divided into two groups: “<16 class hours” and “≥16 class hours.” Table 4 presents the effect sizes from subgroup analyses grouped by course duration. The results showed that interventions with ≥16 class hours had better improvement effects than those with <16 class hours in the dimensions of fear of death (−0.41 vs. −0.21), death avoidance (−0.45 vs. −0.16), and escape acceptance (−0.36 vs. −0.10), indicating that sufficient course duration is crucial for in-depth changes in death attitudes. It should be noted that the “<16 class hours” subgroup only includes 2 studies, so the stability of the conclusion needs further verification.

**Table 4:** Effect sizes of subgroup analysis by course duration.

Course duration	<16 Hours	≥16 Hours
Number of studies	2	10
Sample size	124	987
Fear of death	−0.21 [−0.45, 0.04]	−0.41 [−0.66, −0.17]
*p*-value	0.11	0.00
Death avoidance	−0.16 [−0.40, 0.09]	−0.45 [−0.72, −0.19]
*p*-value	0.22	0.00
Neutral acceptance	0.40 [0.15, 0.66]	0.18 [−0.25, 0.61]
*p*-value	0.00	0.40
Approach acceptance	0.10 [−0.16, 0.36]	0.33 [0.00, 0.66]
*p*-value	0.45	0.05
Escape acceptance	−0.10 [−0.35, 0.14]	−0.36 [−0.67, −0.06]
*p*-value	0.41	0.02

#### 3.4.3 Subgroup by cultural background

Studies were divided into two groups: “China” and “Others”. Table 5 presents the effect sizes from subgroup analyses grouped by cultural background. The “China” group demonstrated greater improvement in neutral acceptance compared to other countries (SMD = 0.29 vs. 0.09), which may be associated with cultural factors (3, 14). These exploratory cultural comparisons require validation through intentionally designed cross-cultural studies before firm conclusions can be drawn.

**Table 5:** Subgroup analysis by cultural background.

Cultural background	China	Others
Number of studies	8	4
Sample size	690	421
Fear of death	−0.42 [−0.67, −0.17]	−0.30 [−0.73, 0.13]
*p*-value	0.00	0.18
Death avoidance	−0.35 [−0.58, −0.13]	−0.51 [−1.10, 0.07]
*p*-value	0.00	0.09
Neutral acceptance	0.29 [0.17, 0.40]	0.09 [−1.07, 1.25]
*p*-value	0.00	0.88
Approach acceptance	0.12 [0.02, 0.23]	0.68 [−0.04, 1.40]
*p*-value	0.02	0.07
Escape acceptance	−0.23 [−0.44, −0.01]	−0.51 [−1.21, 0.20]
*p*-value	0.04	0.16

### 3.5 Sensitivity analysis

**Figure 5:**
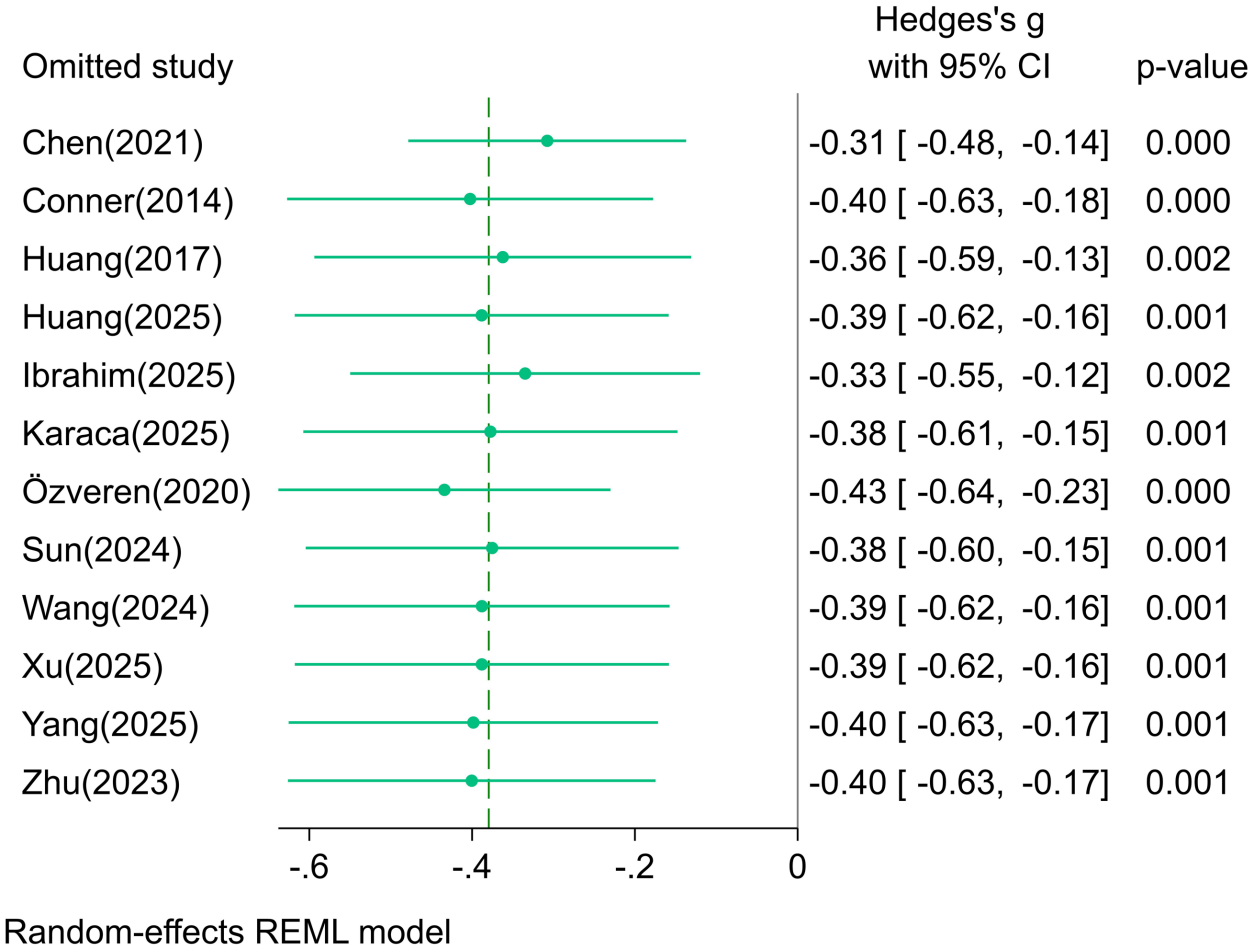
Forest plot of “One-Study-at-a-Time Exclusion” for the fear of death dimension.

Sensitivity analysis was conducted using the “one-study-at-a-time exclusion” method. The forest plot of “one-study-at-a-time exclusion” for the fear of death dimension is shown in Figure 5. The fluctuation ranges of the pooled effect sizes were −0.43 ~ −0.31 (fear of death), −0.46 ~ −0.35 (death avoidance), 0.10 ~ 0.24 (neutral acceptance), 0.17 ~ 0.32 (approach acceptance), and −0.35 ~ −0.24 (escape acceptance), with no significant changes, indicating good stability of the results.

### 3.6 Publication bias

**Figure 6:**
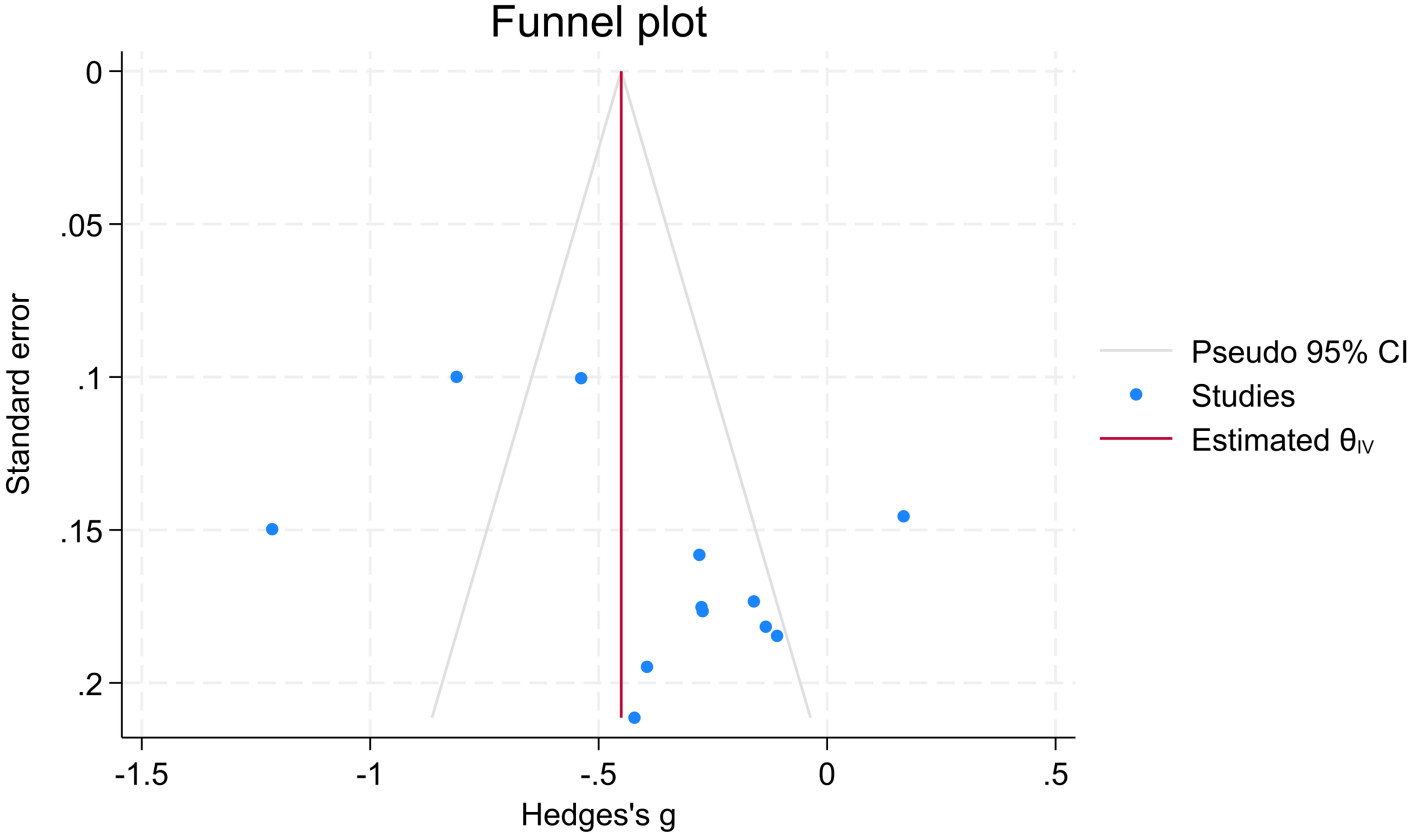
Funnel plot (for the fear of death dimension).

To assess potential publication bias, a funnel plot was drawn in Figure 6 using the fear of death dimension as an example. The results showed that the funnel plot was basically symmetric; Egger’s test indicated no significant publication bias (*p* = 0.27).

## 4 Discussion

### 4.1 Implications of high heterogeneity and statistical vs. clinical significance

The consistently high heterogeneity observed across DAP-R dimensions (I^2^ > 80%) warrants careful interpretation of our findings. While random-effects models appropriately account for this heterogeneity, the wide prediction intervals suggest that the true effect of death education interventions may vary substantially across different implementation contexts. Several factors may contribute to this heterogeneity: (1) variations in intervention intensity and content, (2) cultural differences in death attitudes baseline levels, (3) diverse outcome measurement timepoints, and (4) differences in participant characteristics (e.g., clinical experience, prior exposure to death). Meta-regression was considered to explore moderators (e.g., baseline death anxiety, instructor training, outcome measurement timing) but deemed inappropriate due to the limited number of included studies (only 12) and insufficient statistical power for such an analysis with multiple covariates, which might lead to unstable regression results. Furthermore, it is crucial to distinguish between statistical significance and clinical/educational relevance. While many pooled effect sizes reached statistical significance, their confidence intervals were often wide, and some effects were small. Educators and policymakers should consider not only the statistical evidence but also the practical significance of these improvements in the context of curriculum design and resource allocation.

### 4.2 Core effects and mechanisms of death education interventions

This study shows death education interventions has potential and relatively stable effects on improving medical students’ death attitudes, which is consistent with the conclusions of most existing studies.

#### 4.2.1 From the perspective of death attitude transformation

Meta-analysis shows that death education interventions reduces medical students’ fear of death scores, which aligns with the findings of the four-stage “listening-seeing-touching-transcending” death model (25). Notably, after receiving systematic scientific training, medical students are more inclined to view death as a “natural physiological process” (neutral acceptance). For instance, a Turkish study integrated religious and cultural content, yet medical students’ approach acceptance scores did not increase significantly (15). This indicates that scientific and rational cognition dominates medical students’ death attitudes. Constructivist courses break the “passive acceptance” limitation of traditional education through group sharing and role-playing exercises. This helps internalize the perception that “death is a natural law” into a stable attitude. Furthermore, “physical immersion (lying in a simulated coffin) + emotional resonance (reading simulated elegies)” activates students’ deep emotional experiences. This “embodied cognition” mechanism effectively mitigates the fear caused by death taboos, corroborating the conclusion that “spiritual care enhances death acceptance” (32).

#### 4.2.2 From the perspective of ability improvement

Death education interventions improves medical students’ attitudes toward palliative care, which can be explained by the “situated learning theory.” Real scenarios such as hospice ward internships and nursing home services allow medical students to directly observe patient suffering and family grief. This direct observation triggers “emotional resonance” (31). It is important to note that mastering knowledge alone is insufficient to translate into clinical behavior; instead, “case discussions + simulated practice” must be integrated. This suggests that death education interventions should avoid the tendency of “valuing knowledge over application” (33). Combining standardized patient simulation for communication training with clinical internships helps students master the skills of “breaking bad news.” This approach yields significant intervention effects. Additionally, incorporating “death event reflection sessions” during internships enables students to convert classroom knowledge into practical competence (10). This “practice-reflection-repractice” cycle aligns with the survey result that “students most require ‘real-scenario learning’” (34). Moreover, interprofessional collaborative learning is critical for the future work of multidisciplinary palliative care teams. Examples include joint participation of medical and nursing students in death case discussions (35).

### 4.3 Preliminary evidence for curriculum design: suggested directions for death education

The subgroup analysis results provide preliminary suggestions for the curriculum design of death education for medical students. Core recommendations as follows:

#### 4.3.1 Intervention method: prioritize blended learning and strengthen practical modules

Blended learning (online + offline) showed the associations with improved outcomes in our exploratory analysis. Another study reported a 92.4% satisfaction rate for this blended model, significantly higher than single theoretical teaching (68.3%) (27). Structured courses also demonstrate advantages (36): by “identifying knowledge gaps through pre-tests and consolidating effects through post-tests.” These advantages are reflected in two aspects:

- Online module: adapts to the “heavy curriculum and fragmented time” characteristics of medical students. Theories (e.g., death culture, ethical regulations) can be delivered via MOOCs. Online discussion forums can facilitate interprofessional communication (e.g., nursing and clinical medical students jointly discussing “palliative communication strategies”).
- Offline module: focuses on “immersive experiences” and recommends incorporating three core practical components: (1) Hospice ward internships to learn symptom management and family communication; (2) Funeral home/cemetery visits to understand funeral procedures and the dignity of life; (3) Simulated death experiences, such as “writing epitaphs” and “lying-in-coffin experience,” to strengthen reverence for life.

#### 4.3.2 Intervention duration: ≥16 class hours is promising, balancing “depth” and “feasibility”

Subgroup analysis revealed that interventions lasting ≥16 class hours might be more effective than those with <16 class hours. A promising direction could be to offer death education as a “semester-long course” (e.g., 8 weeks, 2 class hours per week) rather than short-term workshops. The reason is that changing medical students’ emotional attitudes toward death requires a gradual “cognition-emotion-behavior” process. Sufficient time also avoids “information overload” and consolidates effects through “reflection diaries” and “group discussions.”

#### 4.3.3 Content design: integrate student characteristics and cultural background to achieve “localized adaptation”

- Differentiated design by target group: surveys show that junior medical students require more “basic death cognition” education, like the physiological process of death. Senior students, in contrast, focus more on “clinical death response” such as family comfort (37). Nursing students show significantly stronger willingness to learn “palliative care skills” than students majoring in religious education. This difference stems from their professional needs (38). Additionally, male students tend to have lower knowledge scores (2) Therefore, more interactive and practical interventions should be designed for them.
- Culturally localized design: death education should incorporate local cultural elements. Medical students in China are influenced by Confucian culture, which traditionally avoids discussing death. This makes them more prone to death anxiety and avoidance. The “four-stage teaching model” improved medical students’ neutral acceptance scores. It achieved this by integrating culturally relevant content such as Coco (on life inheritance) and Qingming Festival tomb-sweeping traditions (28). In contrast, Islam’s doctrine that “death is a transition of life” fosters higher death acceptance, so interventions can focus on “palliative spiritual care” (39). A Turkish study found that nurses pay more attention to the “emotional impact of death,” so interventions should strengthen modules on “self-care and emotional regulation” (40). These cultural differences indicate that death education cannot adopt a one-size-fits-all model; instead, content should be adjusted based on local cultural values to retain cultural flexibility within a standardized framework.

### 4.4 Implementation challenges and solutions for medical students’ death education

Based on practical feedback from included studies, current death education for medical students faces two major challenges that require targeted solutions:

#### 4.4.1 Challenge 1: insufficient integration of “death education + clinical practice”

Among the included studies, 66.7% of courses were led by nursing/clinical teachers, but only 33.3% of these teachers had received systematic death education training (29, 31). This leads to a tendency of “emphasizing teaching over clinical practice” in courses. One potential strategy to consider would be establishing a “dual-teacher faculty team”: co-taught by “clinical teachers (responsible for care skills)” and “specialized teachers (responsible for emotional counseling).” Meanwhile, an “internship-education linkage mechanism” should be built. This would involve setting up “death event reflection sessions” during clinical internships, where mentors guide students to discuss real death cases and convert classroom knowledge into practical abilities. Promote the “simulation-reality transition model”: first train communication skills through standardized patients, then gradually involve students in real palliative care to reduce psychological impact. Additionally, death education can be included as a reference indicator in medical students’ professional qualification examinations. This would urge institutions to prioritize education quality, ultimately ensuring that “every medical student possesses basic death response and palliative care capabilities.”

#### 4.4.2 Challenge 2: lack of a long-term effect evaluation system

Few studies conducted follow-ups exceeding 3 months. None tracked students’ practical behaviors after entering clinical practice (e.g., whether they proactively participate in palliative care) (41). Furthermore, although attempts were made to explore the impact of education on patient care, evaluations relied solely on students’ self-reports. This approach lacked objective assessments from patients or their families. It is recommended to establish a “three-level tracking system (curriculum-internship-work)” and adopt a mixed evaluation method combining “student behavior observation + patient satisfaction survey.” This approach would include: (1) Assess attitudes and knowledge upon course completion; (2) Evaluate “stress responses when facing patient death” 6 months after clinical internships; (3) Measure “palliative care practice behaviors” 1 year after employment. Long-term data will verify the sustainability of death education effects.

### 4.5 Limitations

- Literature quality and type: included studies were before-and-after controlled trials, and most quasi-experimental studies lacked random allocation and allocation concealment, which may introduce selection bias. All included studies are non-randomized intervention studies, so the evidence level is lower than that of randomized controlled trials, and causal inference should be made with caution.
- Language limitation: only Chinese and English literatures were included, potentially missing high-quality studies in other languages and posing a risk of publication bias.
- Heterogeneity in measurement tools: although the DAP-R scale was used for the primary outcome, some studies adopted different versions (e.g., Chinese, English, Turkish versions), which may cause bias in effect size pooling.
- Sample representativeness: Chinese studies accounted for 66.7% (8/12) of the included literature, so caution is required when extrapolating results to medical students of multiple majors worldwide.

## 5 Conclusion

Based on the evidence from non-randomized intervention studies, death education has potential and relatively stable effects on improving medical students’ death attitudes (reducing fear and avoidance, enhancing neutral acceptance, etc.) and enhancing their palliative care literacy. Blended learning (online theory + offline practice) and an intervention duration of ≥16 class hours represent potentially optimal strategies that warrant further validation through rigorous RCTs. It is suggested that medical colleges and universities incorporate death education into their core curriculum, design “commonality + individuality” course content based on professional characteristics and local culture, and establish “embedded clinical connection” and “long-term tracking evaluation” systems to support the cultivation of high-quality medical talents with “integration of humanity and technology.” Future research should conduct more multicenter studies to explore optimization paths for death education among medical students of different majors and cultural backgrounds.

## Data Availability

The original contributions presented in the study are included in the article/supplementary material, further inquiries can be directed to the corresponding author.
